# Survey on antimicrobial usage in local dairy cows in North-central Nigeria: Drivers for misuse and public health threats

**DOI:** 10.1371/journal.pone.0224949

**Published:** 2019-12-26

**Authors:** Nma Bida Alhaji, Mohammed Baba Aliyu, Ibrahim Ghali-Mohammed, Ismail Ayoade Odetokun

**Affiliations:** 1 Department of Public Health and Epidemiology, Niger State Ministry of Livestock and Fisheries, Minna, Nigeria; 2 Department of Veterinary Public Health and Preventive Medicine, University of Ilorin, Ilorin, Nigeria; Tokat Gaziosmanpasa University, TURKEY

## Abstract

**Background:**

Antimicrobials are used as a measure to maintain good health and productivity of lactating cows. This study assessed pastoralists’ knowledge and practices regarding AMU in lactating cows; and risk pathways for AMR dissemination from cow milk to humans.

**Methods:**

Interview questionnaire-based cross-sectional study was conducted in Fulani pastoral communities. Frequencies and proportions were used for descriptive statistics. Chi-square test and multivariable logistic regressions were used for analytic statistics at 95% confidence level.

**Results:**

All recruited 384 pastoral households participated. About 11% of participants indicated antimicrobials misuse as when given at under-dose, while 58.9% had no knowledge of what antimicrobial misuse entailed. Most participants (51.6%) were unaware about effects of improper AMU. Most respondents (61.7%) reported self-prescription of antimicrobials used on cows. Also, 67.4% of respondents reported arbitrary applications of antimicrobials used in cows, while 15% used antimicrobials to increase milk yield. Frequently used antimicrobials were: tetracycline (98.7%), penicillin (96.6%), streptomycin (95.8%) and sulfonamides (95.3%). Consumption of raw milk and milk products (p = 0.010); contacts with contaminated udder (p = 0.002); and aerosols of discarded contaminated milk P = 0.001) were perceived risk pathways for spread of antimicrobial resistance from cow milk. Improper AMU (p<0.001), non-enforcement of regulating laws (p<0.001), weak financial status (p<0.001), and low education and expertise (p<0.001) influenced antimicrobials misuse in lactating cows.

**Conclusions:**

This study highlighted low levels of knowledge, risk perceptions and practices regarding AMU and AMR among survey pastoralists. This calls for education of the vulnerable populations on promotion of prudent AMU in lactating cows through ‘One Health’ approach, to assure food safety, food security, and public and environmental health.

## Introduction

Milk is an important source of nutrients and most popular natural health food to both humans and animals [1]. Dairy products provide many highly bioavailable essential nutrients, which are especially important in human diets, and even small amounts of dairy products can improve the nutritional status of those living in low-income households [2]. Dairy production is rapidly expanding in developing countries due to population growth [3]. In Nigeria, the annual average national milk consumption is about 20 liters, which is four times less than World Health Organization (WHO) minimum recommendation [4]. One of the major problems of local cattle milk production worldwide is bovine mastitis, which has significant impact on the economy of milk production, due to high cost of antimicrobial agents [5,6].

Annual antimicrobial usage (AMU) in food animals was globally estimated at 63,000 tons in 2015, with projected increase of about 67% by 2030 [7]. Apart from the top consumers of antimicrobials that include China, United States and Brazil, the largest relative increase of more than 200% is projected to occur in developing countries, with Myanmar, Indonesia and Nigeria taking the lead [7]. Improper AMU in food animals contributes to development of antimicrobial resistance (AMR), currently a global health problem and can spread between countries [8–10]. Improper AMU in food animals creates selective evolutionary pressure that enables antimicrobial resistant pathogens to emergence and increase in numbers more rapidly than antimicrobial susceptible pathogens, posing a serious health threat [8].

Fulani pastoralists in Nigeria live in rural areas, herding about 90% of ruminants, relied on the livestock for social and economic well-being and practice nomadic or seasonal transhumance grazing system [11]. These pastoralists commonly use antimicrobials on lactating cows primarily to treat or prevent mastitis and increase milk production or improve feed efficiency. However, because they are domiciled in remote areas, conventional veterinary services for the animals are poor and basic information on AMU is not readily available. [12].

Due to public health and socio-economic impacts of AMR, investigation into risks associated with AMU dynamics in lactating cows becomes imperative. However, the risk of AMR emergence and dissemination through milk to humans can better be assessed within the premise of “Perceived Susceptibility” construct of the Health Belief Model, which postulates that perceived personal risk or susceptibility prompt people to adopt healthier behaviours. The greater the risk perception about a health threat, the greater the likelihood of engaging in behavours that will decrease its risk [13]. Exploration of pastoralists’ local knowledge and practices regarding AMU in lactating cows is, therefore, crucial for the development of effective AMR surveillance, control and prevention. Study objectives were to: 1) assess pastoralists’ knowledge and practices regarding AMU and AMR in lactating cows; and 2) assess risk pathways for AMR dissemination from lactating cows to humans. In addition, we sought to explore socio-demographic determinants of knowledge on AMU and resistance, and socio-economic activities that influence antimicrobials misuse and resistance emergence in lactating cows.

## Materials and methods

### Structure of target population and livelihood

Target populations were households in Fulani pastoral communities, who are seasonally mobile with herds of local breeds of cattle, domiciled in the study area during the survey. Each pastoral community had an average 28 households deriving their livelihoods mainly from sales of milk and milk products. A Fulani pastoral household constituted a herd managed by herd head or owner. Average number of cattle in a herd was 82 animals, with ratio of 1:10 bull to cows. Study eligibility was based on a participant being a household head and at least 20 years of age. They are expected at these ages to possess existing veterinary knowledge on livestock health and production management because of long time intimate relationship with the animals.

### Study design, sample size and sampling procedure

An interview questionnaire-based cross-sectional survey was conducted in randomly selected pastoral households in Fulani nomadic pastoral communities of North-central Nigeria in 2017. The sample size was calculated using the Open Source Epidemiologic Statistics for Public Health (OpenEpi) 2.3.1 software [14] for percentage frequency in a population (random sample). The following assumptions were used: finite population because the target population was not large and mobile in nature, 50% expected frequency of respondents that use antimicrobials on cows, a desired absolute precision set at 5%, and 95% confidence interval. Based on these assumptions, a sample size of 384 pastoralists’ households was obtained. A two-stage sampling method was used. In the first stage, 30 Fulani pastoral communities were purposively selected across the study area. In the second stage, systematic random sampling was used to select all targeted households.

### Questionnaire design, pretesting and data collection

A structured questionnaire (S1 Questionnaire) that contained mostly close-ended questions, to ease data processing and improve response precision [15], was designed based on experts’ opinions. It consisted of five sections: i) pastoralist’s socio-demographic characteristics (6 questions); ii) knowledge about AMU in lactating cows: antimicrobials misuse, AMR and its effects in animals and humans (13 questions); (iii) practices of AMU in cows (13 questions); (iv) risk pathways for antimicrobial resistance dissemination (8 questions); and (v) factors that influence antimicrobials misuse and resistance emergence in cows (6 questions). The questionnaire was designed in English and verbally translated into local *Hausa* languages during administration, for those without formal education. They were asked questions in *Hausa* and responses translated to English during recording. Six animal health officials were trained to administer the questionnaires, pre-tested on households in a community before final administration, to identify problems and eliminate them for adequate data delivery.

Respondents were provided with verbal information on objectives of the study. Their informed consent was verbally obtained with signatures and thumbs printing on a sheet before questionnaire administration and none declined to participate. They were assured of voluntary participation, confidentiality of responses and the opportunity to withdraw at any time without prejudice in line with the World Medical Association Declaration of Helsinki [16]. Verbal information and informed consent were deemed necessary because of low literacy levels among participants. Advocacy visits were made to each pastoral community a week prior to the proposed interview and necessary permission obtained from ‘*Ardos*’ (leaders).

### Ethics statement

The Research Ethics Committee of Niger State Ministry of Livestock and Fisheries, Minna, Nigeria approved the research proposal (Ref #. MLF/NGS/693).

### Data management and statistical analysis

Participants’ responses were first summarized into Microsoft Excel 7 (Microsoft Corporation, Redmond, WA, USA) spreadsheets. Descriptive statistics were calculated for all variables in forms of frequencies and proportions, and associations between variables were determined by Chi-square test or Fischer’ exact test where appropriate, and by multivariable logistic regressions models.

The participants' level of knowledge was determined according to outcome criteria previously identified by Alhaji et al. [17]: the word “very low” represented a proportion of respondents with “know” knowledge that ranged from 1% to 24%; “low” represented proportion with “know” knowledge that ranged between 25% and 49%; “high” represented proportion with “know” knowledge that ranged between 50% and 74%; and “very high” represented proportion with “know” knowledge that ranged from 75% to 100%. Similar approach was used to identify levels of practices and perceptions. To assess association, independent (explanatory) variables were created from the socio-demographic characteristics and socio-economic factors that influence antimicrobial misuse, while respondents' overall response levels constituted the dependent (outcome) variables. The outcome variables were coded by a unique scoring system, with a response score that ranged between 1 and 20 points and converted to 100%. The score range was further categorized into ‘poor’ or ‘satisfactory’ to keep them in categorical forms. Response scores within 1–10 points were considered ‘poor’ (≤49%), and those within 11–20 points were considered ‘satisfactory’ (≥50%).

Associations between explanatory and outcome variables were first subjected to univariable analysis using Chi-square tests [18]. Factors found to be statistically significant at this analysis were finally subjected to likelihood stepwise backward multivariable logistic regressions models to control for confounding and test for effect modification. All statistical analyses were conducted using EpiInfo 3.4.3 (CDC, Atlanta, GA, USA) and OpenEpi version 2.3.1 [14]. A p<0.05 was considered statistically significant in all analyses.

## Results

### Socio-demographic characteristics of participants

**Fig 1:**
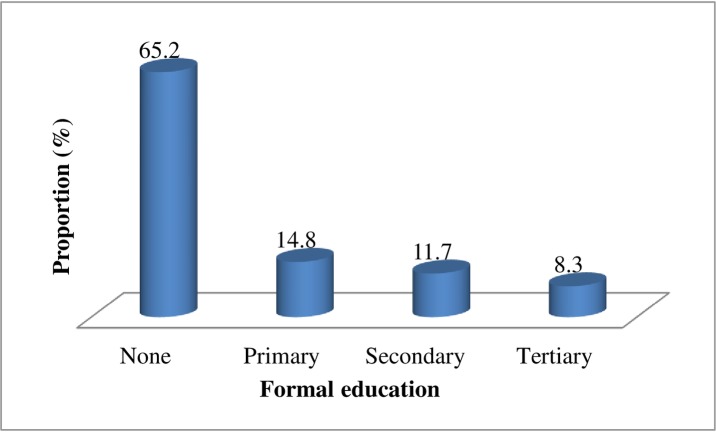
Formal educational status of Fulani pastoralists in North-central Nigeria.

All recruited 384 Fulani pastoralists participated in the study. Most of the participants (25.0%, 96/384) were in age group 50–59 years. Majority the respondents were males (84.4%, 328/384) and married 84.4%, while 8.6% (33/384) were single and 7.0% (27/384) widows. The majority of pastoralists (65.2%, 250/384)) had no formal education and only 8.3% (32/384) had tertiary education (Fig 1).

### Knowledge about AMU and antimicrobial resistance in lactating cows

Most respondents (53.4%) believe that antimicrobials are used to treat mastitis in lactating cows, 24.2% to prevent mastitis in lactating cows, and 15.4% to promote production of milk in lactating cows, while 7.0% of participants indicated knowing antimicrobials to be used in cows for all of the above.

Also, only 10.9% of participants knew antimicrobials misuse in cows to be when given as under-dose, 10.2% mentioned when given at over-dose and 58.9% did not know what misuse entailed. Regarding whether antimicrobials misuse in cows can predispose to the emergence of resistance, 26.8% of pastoralists agreed and 41.1% disagreed. On AMR effects, 51.6% and 60.4% of participants did not know the consequences in lactating cows and humans, respectively. Regarding dissemination of AMR from lactating cows to humans, only 13.0% of respondents mentioned drinking raw milk, 12.2% reported drinking fermented milk (*nono*), while 8.9% indicated milking cows and 60.4% had no idea (Table 1).

**Table 1:** Fulani pastoralists' knowledge about AMU and resistance in lactating cows in North-central Nigeria.

Variable	Frequency (n)	Proportion (%)	Confidence interval
**Know antimicrobials to be used**			
To treat mastitis in lactating cows	205	53.4	48.3858.34
To prevent mastitis in lactating cows	93	24.2	20.13, 28.69
To promote milk yield in lactating cows	59	15.4	12.02, 19.23
All of the above	27	7.0	4.78, 9.93
**Antimicrobials misuse in lactating cows is when**			
Administered under-dose	42	10.9	8.10, 14.36
Administered over-dose	39	10.2	7.43, 13.49
Administered in normal dose	77	20.0	16.27, 24.28
Don’t know	226	58.9	53.87, 63.7
**Effects of antimicrobials misuse on lactating cows**			
Non response to bacterial infection treatment	121	31.5	27.01, 36.29
Extra costs on treatment of bacterial infection	65	16.9	13.42, 20.93
Don’t know	198	51.6	46.56, 56.54
**Antimicrobials misuse in lactating cows can predispose to resistance emergence**			
Agree	103	26.8	22.57, 31.42
Disagree	158	41.1	36.3, 46.13
Don’t know	123	32.1	27.51, 36.83
**Antimicrobial resistance can be passed from lactating cows to humans through**			
Drinking raw milk	50	13.0	9.93, 16.67
Drinking fermented milk (*nono*)	47	12.2	9.24, 15.81
Eating raw cheese (*wara*)	21	5.5	3.51, 8.10
Milking cows	34	8.9	6.31, 12.02
Don’t know	232	60.4	55.46, 65.22
**Effects of antimicrobial resistance in humans**			
Non response to bacterial infection treatment	56	14.6	11.32, 18.38
Extra costs on treatment of bacterial infection	41	10.7	7.88, 14.07
Longer duration of illness and treatment	55	14.3	11.08, 18.1
Don’t know	232	60.4	55.46, 65.22

### Practices of AMU on lactating cows

On personnel that prescribed antimicrobials for usage in lactating cows, 28.1% of the pastoralists reported animal health officials, while 61.7% engaged in self-prescription. More than one-third (34.9%) of the participants purchased antimicrobials used on cows from veterinary drug shops; 10.7% obtained the drugs from human drug shops, while 54.4% patronized animal drug hawkers. Also, 65.4% of participants practiced self-administration of antimicrobials used on cows, and 32.6% engaged services of animal health officials. Regarding rate of AMU on lactating cows with mastitis, 19.3% of respondents reported following prescribed instructions, 27.1% administered antimicrobials only once on sick cows until they recovered, while 53.6% administered antimicrobials once daily on sick animals until recovered.

On dosage determination before usage on cows, 34.6% of the pastoralists reported following instructions on labels, and 65.4% mentioned arbitrary applications. Majority of the pastoralists (57.6%) frequently administered antimicrobials by injection and very few of them (3.6%) applied the drugs through feed. However, 18.7% of respondents observed withdrawal periods after AMU on cows, and most of them (81.3%) reported non-compliance with withdrawal periods. On purpose for AMU on lactating cows, most respondents (53.4%) mentioned that they used antimicrobials to treat mastitis in lactating cows, 24.2% to prevent mastitis in lactating cows, and 15.4% to promote milk production in lactating cows (Table 2).

**Table 2:** Practices of antimicrobial usage on lactating cows by Fulani pastoralists in North-central Nigeria.

Practice	Frequency (n)	Proportion (%)	Confidence interval
**Personnel that prescribe antimicrobials for usage in lactating cows**			
Animal health officials	108	28.1	23.8, 32.78
Self-prescription	237	61.7	56.78, 66.48
Friends and relations	39	10.2	7.43, 13.49
**Purchasing places of antimicrobials**			
Veterinary drug shops	134	34.9	30.25, 39.77
Human drug shops	41	10.7	7.88, 14.07
Animal drug hawkers	209	54.4	49.42, 59.37
**Who administer antimicrobials on cows**			
Self-administered	259	67.4	62.64, 72.00
Animal health officials	125	32.6	28.00, 37.36
**Frequency of antimicrobial usage on lactating cows with mastitis**			
As prescribed	74	19.3	15.56, 23.45
Only once	104	27.1	22.82, 31.7
Once daily until recovered	206	53.6	48.64, 58.6
**Dosage determination before antimicrobials use**			
From instructions on the label	133	34.6	30.00, 39.50
Arbitrary	251	65.4	60.50, 70.00
**Frequently used route of administration**			
Injection	221	57.6	52.56, 62.43
Mouth (POS)	101	26.3	22.08, 30.88
On the skin (topical)	48	12.5	9.47, 16.10
In feed	14	3.6	2.09, 5.90
**Observe about withdrawal periods**			
Yes	72	18.7	15.08, 22.89
No	312	81.3	77.11, 84.92
**Purpose for antimicrobial usage**			
Treatment of mastitis in lactating cows	205	53.4	48.38, 58.34
Prevention of mastitis in lactating cows	93	24.2	20.13, 28.69
Promotion of milk yield in lactating cows	59	15.4	12.02, 19.23
All of the above	27	7.0	4.78, 9.93

### Antimicrobials frequently used on lactating cows

**Fig 2:**
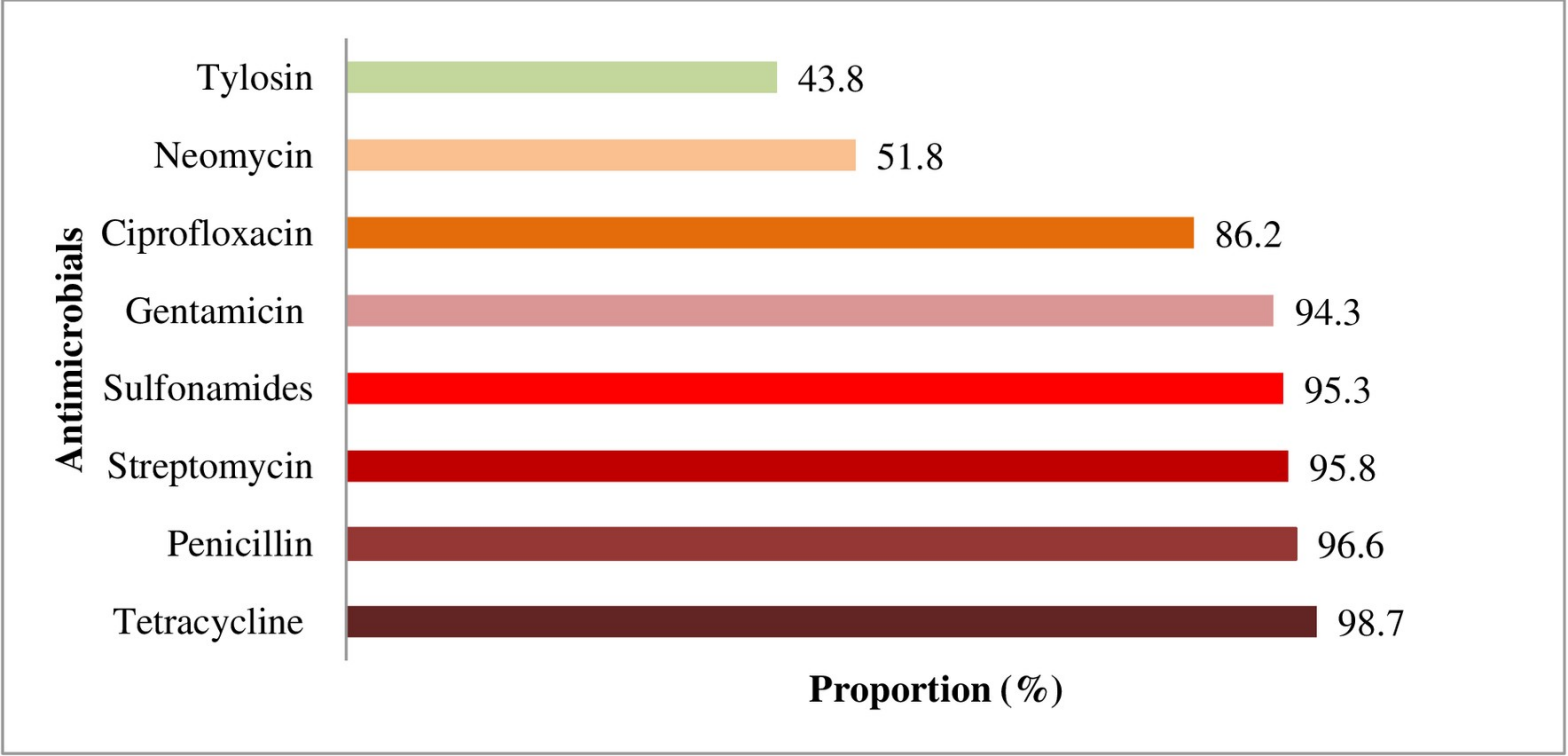
Antimicrobials frequently used by Fulani pastoralists on lactating cows in North-central Nigeria.

Pastoralists recounted usage of a range of different classes of antimicrobials on lactating cows. Most frequently used antimicrobials were: tetracycline (98.7%), penicillin (96.6%), streptomycin (95.8%), sulfonamides (95.3%), gentamicin (94.3%), ciprofloxacin (86.2%) and neomycin (51.8%) (Fig 2).

### Perceptions on risk pathways for antimicrobial resistance dissemination from cow milk to humans

Fulani pastoralists significantly perceived variably low, moderate and high risks associated with pathways for transmission of antimicrobial resistance from cow milk to humans. Few pastoralists perceived consumption of contaminated raw milk (14.8%), fermented milk (10.1%), and raw cheese (9.3%) to be of high risk for spread of antimicrobial resistance to humans. Also, very few of the participants perceived contacts with contaminated udder and milk (13.3%), and fomites (6.2%) to be of high risk for dissemination of resistance from cow milk to humans. Furthermore, very few participants perceived aerosols of discarded contaminated milk (18.0%), milking of cows in the herd site (10.4%), and flies attracted to the contaminated milk in the site (7.5%) to be high risk pathway for dissemination of AMR from cow milk to humans through environment (Table 3).

**Table 3:** Pastoralists’ perceptions on risk pathways for antimicrobial resistance dissemination from cow milk to humans in North-central Nigeria.

Risk pathway	Low risk n (%)	Moderate risk n (%)	High risk n (%)	Chi-square	P-value
**Consumption of raw milk and milk products**				**11.98**	**0.010**
Raw milk	225 (58.6)	102 (26.6)	57 (14.8)		
Fermented milk (*nono*)	221 (57.6)	124 (32.3)	39 (10.1)		
Raw cheese (*wara*)	251 (65.4)	97 (25.3)	36 (9.3)		
**Direct or indirect contacts**				**11.66**	**0.002**
Contacts with contaminated udder and milk	243 (63.3)	90 (23.4)	51 (13.3)		
Contacts with contaminated fomites	251 (65.4)	109 (28.4)	24 (6.2)		
**Environment**				**21.22**	**0.001**
Aerosols of discarded contaminated milk	229 (59.6)	86 (22.4)	27 (18.0)		
Aerosols during milking of cows	253 (65.9)	120 (31.2)	11 (2.9)		
Flies attracted to contaminated milk	279 (72.7)	76 (19.8)	29 (7.5)		

Low risk (< 35%); Moderate risk (35–65%); High risk (> 66%); Significant at p<0.05

### Socio-demographic characteristics associated with knowledge on AMU in cows

Results of multivariable logistic regressions indicated that Fulani pastoralists in age group 70–79 years were five times more likely to possess satisfactory knowledge about AMU in lactating cows than those in age group 20–29 years (OR = 4.56; 95% CI: 1.89, 11.02; p = 0.001). Also, male pastoralists were more likely to possess satisfactory knowledge about AMU in cows than females (OR = 3.10; 95% CI: 1.69, 5.65; p = 0.001). Meanwhile, pastoralists with tertiary education were four times more likely to possess satisfactory knowledge about AMU in lactating cows than those without formal education (OR = 4.21; 95% CI: 1.82, 9.74; p = 0.001) (Table 4).

**Table 4:** Socio-demographic characteristics of Fulani pastoralists associated with knowledge regarding AMU on lactating cows in North-central Nigeria.

Variable	Number of respondents n (%)	Poor knowledge n (row %)	Satisfactory knowledge n (row %)	Odds ratio (OR)	95% Confidence interval	P-value
**Age (in years)**						
20–29	44 (11.5)	29 (65.9)	15 (34.1)	1.00		
30–39	56 (14.5)	33 (58.9)	23 (41.1)	1.35	0.59, 3.06	0.485
40–49	69 (18.0)	32 (46.4)	37 (53.6)	2.24	1.02, 4.89	0.040
50–59	96 (25.0)	40 (41.7)	56 (58.3)	2.71	1.29, 5.69	0.008
60–69	72 (18.8)	26 (36.1)	46 (63.9)	3.42	1.56, 7.52	0.002
70–79	47 (12.2)	14 (29.8)	33 (70.2)	4.56	1.89, 11.02	0.001
**Gender**						
Female	56 (14.6)	38 (67.9)	18 (32.1)	1.00		
Male	328 (84.4)	133 (40.5)	195 (59.5)	3.10	1.69, 5.65	0.001
**Formal education**						
None	250 (62.2)	146 (58.4)	104 (41.6)	1.00		
Primary	57 (14.8)	22 (38.6)	35 (61.4)	2.23	1.24, 4.03	0.007
Secondary	45 (11.7)	19 (42.2)	26 (57.8)	1.92	1.01, 3.65	0.040
Tertiary	32 (8.3)	8 (25.0)	24 (75.0)	4.21	1.82, 9.74	0.001

Statistically significant at p<0.05

### Socio-economic factors that influence antimicrobials misuse and resistance emergence in lactating cows

Many factors were identified to influence antimicrobials misuse on lactating cows, and which can predispose to emergence and dissemination of antimicrobial resistant pathogens through cow milk to humans. On multivariable logistic regressions models, improper AMU was more likely to satisfactorily influenced antimicrobials misuse on lactating cows (OR: 26.20; 95% CI: 13.79, 49.59; p<0.001). Also, non-enforcement of laws regulating AMU was more likely to influenced antimicrobials misuse in cows (OR: 5.32; 95% CI: 3.35, 8.25; p<0.001). Pastoralists’ weak financial status (OR: 6.73; 95% CI: 4.20, 10.62; p<0.001) and low education and expertise (OR: 10.41; 95% CI: 6.37, 16.87; p<0.001) were more likely to satisfactorily influenced their misuse of antimicrobials on lactating cows. Furthermore, pastoralists’ nomadic culture (OR: 9.75; 95% CI: 5.47, 17.38; p<0.001) and extensive management system of herds (OR: 18.40; 95% CI: 10.97, 30.86; p<0.001) were more likely to influenced antimicrobials misuse on lactating cows (Table 5).

**Table 5:** Socio-economic factors that influence antimicrobials misuse by pastoralists and resistance emergence in lactating cows in North-central Nigeria.

Factor	Poor influence (%)	Satisfactory influence (%)	Odds ratio (OR)	95% CI	P-value
**Improper antimicrobial usage**					
No	111 (63.4)	64 (36.6)	1.00		
Yes	13 (6.2)	196 (93.8)	26.20	13.79, 49.59	<0.001
**Non-enforcement of laws regulating antimicrobial usage**					
No	98 (57.6)	72 (42.4)	1.00		
Yes	44 (20.6)	170 (79.4)	5.32	3.35, 8.25	<0.001
**Weak financial status of pastoralists**					
No	93 (60.0)	62 (40.0)	1.00		
Yes	42 (18.3)	187 (81.7)	6.73	4.20, 10.62	<0.001
**Low education and expertise of pastoralists**					
No	97 (66.9)	48 (33.1)	1.00		
Yes	39 (16.3)	200 (83.7)	10.41	6.37, 16.87	<0.001
**Nomadic and transhumance culture of pastoralists**					
No	55 (73.3)	20 (26.7)	1.00	5.47, 17.38	<0.001
Yes	68 (22.0)	241 (78.0	9.75		
**Extensive husbandry system**					
No	151 (77.0)	45 (23.0)	1.00		
Yes	29 (15.4)	159 (85.6)	18.4	10.97, 30.86	<0.001

Statistically significant at p<0.05

## Discussion

This study, to our knowledge, was the first to survey AMU and AMR in lactating cows, and consequent dissemination from cow milk to humans in rural pastoral communities in Nigeria. It provides critical information regarding antimicrobials stewardship among local smallholders’ dairy farmers under extensive husbandry system. Antimicrobials misuse and resistance in food animals and humans have been recognized as emerging global threat to food safety, food security and public health [19].

This study identified low level of knowledge about proper AMU on lactating cows among surveyed Fulani pastoralists. This could be due to their low literacy level, since 65.2% of them do not possessed formal education. Lack of formal education could influence antimicrobials misuse, which can in turns predispose to residues in milk following treatment of clinical mastitis, a major public health threat with adverse effects on safety of milk for human consumption. Human consumption of milk contaminated with antimicrobials can results in allergic responses, carcinogens, changes in intestinal flora and development of antimicrobial resistant pathogens [20,21]. Knowledge about patterns of AMU is very vital to understanding animal health management in dairy farms. Bridging the knowledge gap through sensitization will increase pastoralists’ risk perceptions associated with AMR in lactating cows.

We found the majority of pastoralists practicing self-prescription (61.7%) and self-administration (67.4%) of antimicrobials on lactating cows without professionals’ inputs. This could be due to gross inadequate veterinary services associated with hard-to-reach terrains of Fulani pastoral communities. In Nigeria, the use of non-prescribed antimicrobials in food animals has been related to unavailability of veterinary services and extra cost of veterinary services [22]. Most of the pastoralists (54.4%) purchase antimicrobials used on cows from animal drugs hawkers without prescriptions, thus facilitating antimicrobials misuse and resistance emergence. Also, antimicrobials were found to be used for therapeutic and preventive purposes as well as for milk yield promotion in lactating cows. This is consistent with findings that reported similar uses in food animals [23,24,25]. Also, improper AMU in food animals has been found to be significant contributor to emergence and dissemination of antimicrobial resistance to humans [26,27]. Noteworthy is our finding on practices of low dose AMU in lactating cows. Antimicrobials at low dosages (subtherapeutic dosages) have been reported to predispose to the resistance emergence in exposed bacteria due to selection bias from promotion of genetic and phenotypic variability [28,29].

This study found some antimicrobials to be frequently used on lactating cow, which include: tetracycline, penicillin, streptomycin, sulfonamides, gentamicin, ciprofloxacin, neomycin and tylosine. This is consistent with previous studies that reported these antimicrobials to be frequently used in food animals in Nigeria [30,31]. About half of the world's antimicrobials productions have been reported to be frequently used in food animals [32,33]. Frequent use of antimicrobials in food animals has been reported in other African countries (South Africa and Tanzania), where tetracycline, sulfonamides, penicillin and streptomycin were mostly used in livestock [32,34]. Similar levels of AMU have been reported in India, where penicillin and tetracycline were frequently used in food animals [35]. Mastitis in lactating cows in pastoral communities was commonly treated with oxytetracycline and gentamicin, which are critically important antimicrobials for human medicine but not approved for use in dairy cattle in the United States due to public health implications [36].

Very few surveyed pastoralists perceived pathways for dissemination of antimicrobial resistance from cow milk to humans to be of high risks. These were through: consumption of contaminated raw milk and milk products (cheese, fermented milk); contacts with contaminated udder and fomites; and contaminated environment (aerosols of contaminated milk). This finding is in consonance with the results that reported transmission of antimicrobial resistance from food animals to humans through food consumption, contact with contaminated animals, and associated wastes spilled into the environment [37,38]. Also, reports have indicated that drug-resistant strains of food animal origin can spread to humans either through food supply chain (dairy products); direct animal contact; or through environment [39,40,41].

We found socio-demographic characteristics of age, gender and education to have significant influence on antimicrobials misuse and emergence of resistance in lactating cows. Health education of pastoralists through mass media, especially radio, is imperative for modification of behaviours and effective social change towards proper AMU on lactating cows in hard-to-reach smallholder pastoral rural settlements in developing economies, especially in Africa.

The study found some socio-economic activities of improper AMU, non-enforcement of laws regulating AMU, weak financial status, low education and expertise, nomadic culture, and extensive husbandry system of Fulani pastoralists to significantly influenced antimicrobials misuse and AMR emergence on lactating cows. Improper AMU has been reported as the major driver of AMR in food animals due to selection pressure on animal microbiota [42]. Improper AMU and lack of enforcement of regulating laws are major factors contributing to increase AMR [43]. Although most of the antimicrobials used by pastoralists were within the categories of the OIE List allowed [44], the major problems were low levels of knowledge and practices on usage. These observed interconnected factors that driven antimicrobials misuse could create complex challenges associated with AMR in cows with public health consequences.

Major limitation of this study was the use of questionnaire tool for data collection. In this case, we pre-tested the questionnaire for quality control and ensured that no information was lost in the process. Also, we were limited by lack of full adjustments for community clustering in the designed random sampling. However, the used of central tendency measures, would be valuable enough to tolerate the likely imperfections in the confidence intervals.

## Conclusions

This study highlighted low levels of knowledge, risk perceptions and practices regarding AMU and AMR among surveyed pastoralists. These challenging gaps, influenced by socio-demographic factors, call for education of the vulnerable populations on promotion of prudent AMU in lactating cows. Consideration of the risk pathways for antimicrobial resistance dissemination is crucial for the development of AMR surveillance system, control and prevention. Gradual reform of socio-economic activities that influence antimicrobials misuse and resistance emergence through collaborations, in line with ‘One Health’ approach, will assure food safety, food security, and public health.

## Supporting information

S1 Questionnaire Tool for gathering data from Fulani pastoralists in North-central Nigeria. (DOCX)

S1 Table Socio-demographic characteristics of Fulani pastoralists in North-central Nigeria. (DOCX)
