# Supplementary material for: Survey on antimicrobial usage in local dairy cows in North-central Nigeria: Drivers for misuse and public health threats
Source: PLoS One. 2019 Dec 26;14(12):e0224949. doi: 10.1371/journal.pone.0224949 (PMC6932773; doi:10.1371/journal.pone.0224949)
Supplement: S1 Questionnaire — (DOCX) [file pone.0224949.s001.docx]

**QUESTIONNAIRE**

This questionnaire is meant to assess pastoralists’ existing knowledge and practices towards antimicrobial usage on lactating cows in cattle herds of North-central Nigeria and you have been identified to participate. Participation is voluntary and all information given will be kept strictly confidential. *PLEASE* ***tick one or more*** *appropriately and* ***write*** *where necessary****.***

Name of interviewer:_______________________ Herd Name:___________________

1. **Demographic Information**

A1. Name of interviewee: ____________________________

A2. Age (in years):_____________

A3. Gender: a. Male ( ) b. Female ( )

A4. Tribe: _________________

A5. Marital status: a. Married ( ) b. Single ( ) c. Widow ( )

A6. Highest formal education:

1. None ( ) b. Primary ( ) c. Secondary ( ) d. Tertiary ( )
2. **Knowledge about Antimicrobial usage in Lactating cows**

B1. Have you ever heard about antimicrobial drugs (*Maganin zazabin dabbobi*)? Yes ( ) No ( )

B2. If Yes, from what source?

1. Radio ( ) b. Friends ( ) c. Relations ( ) d. Animal health authorities ( )

B3. Do you know what antimicrobials are used for on lactating cows? Yes ( ) No ( )

B4. If Yes, what are they used for in lactating cows? a. To treat mastitis (udder infections) ( ) b. To prevent mastitis (udder infections) ( ) c. To increase milk yield ( ) d. All of the above ( )

B5. Do you know what antimicrobials misuse indicates in lactating cows? Yes ( ) No ( )

B6. If Yes, What is it? a. When given under-dose ( ) b. When given over-dose ( ) c. When given in normal dose ( ) d. Don’t know ( )

B7. Do you know the effect(s) of antimicrobials misuse on lactating cows? Yes ( ) No ( )

B8. If Yes, what is/are the effect(s)? a. Non response to treatment of udder infections ( ) b. Extra cost on treatment of infections ( ) c. Don’t know ( )

B9. Antimicrobials misuse on lactating cows can predispose to resistance emergence. a. Agree ( ) b. Disagree ( ) c. Don’t know ( )

B10. Can antimicrobial resistance in lactating cows be passed to humans through food chain? Yes ( ) No ( )

B11. If Yes, through what type of chain? a. Drinking of raw milk ( ) b. Drinking of fermented milk (*nono*) ( ) c. Eating of raw cheese (*wara*) ( ) d. Milking of lactating cow ( ) e. Don’t know ( )

B12. Do you know the effects of antimicrobial resistance in humans? Yes ( ) No ( )

B13. If Yes, what are the effects? a. Non response to treatment of bacterial infections ( ) b. Extra cost on treatment of infections ( ) c. Longer duration of illness and treatment ( ) d. Don’t know ( )

1. **Practices regarding Antimicrobial usage in Lactating cows**

C1. Do you use antimicrobials to treat infected lactating cows in your herd? ­­­­ Yes ( ) No ( )

C2. Who prescribed antimicrobials used on lactating cows for you? a. Animal health personnel ( ) b. Self prescription ( ) c. All of the above ( )

C3. Where do you usually buy the antimicrobials from? a. Veterinary drug shops ( ) b. Human drug shops ( ) c. Animal drug hawkers ( )

C4. Who administer antimicrobials used on the cows in your herd? a. Self administer ( ) b. Animal health officials ( ) c. All of the above ( )

C5. How is antimicrobial dosage determined per cow? a. From instructions on the label ( ) b. Arbitrary ( )

C6. What is the frequency of daily antimicrobial usage on lactating cows? a. As prescribed ( ) b. One single high dose ( ) c. Once daily until the cows recovered ( ) d. Twice daily until the cows recovered ( )

C7. What common disease condition(s) of lactating cows do you use antimicrobials for in your herd? a. Mastitis ( ) b. FMD ( ) c. Udder injuries ( ) d. Others (please specify)………………………

C8. What route(s) do you use for administering antimicrobials in lactating cows? a. By injection ( ) b. POS ( ) c. Through feed ( ) d. All of the above ( )

C9. Do you observe withdrawal period for any antimicrobial administered on lactating cows before consumption of their milk and/or products? Yes ( ) No ( )

C10. What purpose(s) do you administered antimicrobials for on lactating cows? a. To treat infections ( ) b. To prevent infections ( ) c. To increase milk yield ( ) d. All of the above ( )

C11. Have you ever experience antimicrobial resistance in sick lactating cows? Yes ( ) No ( )

C12. If Yes, what are the experiences? a. Non response to treatment ( ) b. Extra cost on treatment of infected cows ( ) c. Longer duration of treatment ( ) d. All of the above ( )

C13. Name antimicrobials you most frequently used on infected lactating cows: .………………………………………………………………………………………………………………………………………………………..

1. **Risk status for Pathways of Antimicrobial resistance Dissemination from Cow milk to Humans**

What are the risk statuses of the following pathways for antimicrobial resistance spread from lactating cows to humans?

D1. Consumption of raw milk: Low risk ( ); Moderate risk ( ); High risk ( )

D2. Consumption fermented raw milk (*nono*): Low risk ( ); Moderate risk ( ); High risk ( )

D3. Consumption of raw cheese (*wara*): Low risk ( ); Moderate risk ( ); High risk ( )

D4. Contacts with contaminated udder and milk: Low risk ( ); Moderate risk ( ); High risk ( )

D5. Contacts with contaminated fomites: Low risk ( ); Moderate risk ( ); High risk ( )

D6. Discarded contaminated milk in the environment: Low risk ( ); Moderate risk ( ); High risk ( )

D7. Aerosols during milking of cows in the environment: Low risk ( ); Moderate risk ( ); High risk ( )

D8. Flies attracted to contaminated milk in the environment: Low risk ( ); Moderate risk ( ); High risk ( )

1. **Factors that Influence Antimicrobials misuse and Resistance Emergence**

What are the factors that influence antimicrobials misuse and resistance emergence in lactating cows?

E1. Improper antimicrobial usage: Yes ( ) No ( )

E2. Non-enforcement of laws regulating antimicrobial usage: Yes ( ) No ( )

E3. Weak financial status: Yes ( ) No ( )

E4. Low formal education and expertise: Yes ( ) No ( )

E5. Nomadic and transhumance culture: Yes ( ) No ( )

E6. Extensive husbandry management system: Yes ( ) No ( )

Thank you for the kind responses

**For further information, contact:**

**E-mail:** [nmabida62@gmail.com](mailto:nmabida62@gmail.com); **Phone (gsm):** +234 (0)803 595 0915
