# Supplementary material for: Survey on antimicrobial usage in local dairy cows in North-central Nigeria: Drivers for misuse and public health threats
Source: PLoS One. 2019 Dec 26;14(12):e0224949. doi: 10.1371/journal.pone.0224949 (PMC6932773; doi:10.1371/journal.pone.0224949)
Supplement: S1 Table — (DOCX) [file pone.0224949.s002.docx]

**Table 1.** Socio-demographic characteristics of Fulani pastoralists in North-central Nigeria

| **Variable** | **Category** | **Frequency**  **(n)** |
| --- | --- | --- |
| **Age (in years)** |  |  |
|  | 20 – 29 | 44 |
|  | 30 – 39 | 56 |
|  | 40 – 49 | 69 |
|  | 50 – 59 | 96 |
|  | 60 – 69 | 72 |
|  | 70 – 79 | 47 |
| **Gender** |  |  |
|  | Female | 56 |
|  | Male | 328 |
| **Marital status** |  |  |
|  | Single | 33 |
|  | Married | 324 |
|  | Widow | 27 |
| **Formal education** |  |  |
|  | None | 250 |
|  | Primary | 57 |
|  | Secondary | 45 |
|  | Tertiary | 32 |

n = number of respondents
